# Integrated genome-wide association, coexpression network, and expression single nucleotide polymorphism analysis identifies novel pathway in allergic rhinitis

**DOI:** 10.1186/1755-8794-7-48

**Published:** 2014-08-02

**Authors:** Supinda Bunyavanich, Eric E Schadt, Blanca E Himes, Jessica Lasky-Su, Weiliang Qiu, Ross Lazarus, John P Ziniti, Ariella Cohain, Michael Linderman, Dara G Torgerson, Celeste S Eng, Maria Pino-Yanes, Badri Padhukasahasram, James J Yang, Rasika A Mathias, Terri H Beaty, Xingnan Li, Penelope Graves, Isabelle Romieu, Blanca del Rio Navarro, M Towhid Salam, Hita Vora, Dan L Nicolae, Carole Ober, Fernando D Martinez, Eugene R Bleecker, Deborah A Meyers, W James Gauderman, Frank Gilliland, Esteban G Burchard, Kathleen C Barnes, L Keoki Williams, Stephanie J London, Bin Zhang, Benjamin A Raby, Scott T Weiss

**Affiliations:** 1 Department of Genetics and Genomic Sciences and Icahn Institute for Genomics and Multiscale Biology, Icahn School of Medicine at Mount Sinai, 10029 New York, NY, USA; 2 Division of Pediatric Allergy and Immunology, Department of Pediatrics, and Mindich Child Health and Development Institute, Icahn School of Medicine at Mount Sinai, New York, NY, USA; 3 Channing Division of Network Medicine, Department of Medicine, Brigham & Women’s Hospital and Harvard Medical School, Boston, MA, USA; 4 Department of Medicine and Department of Bioengineering and Therapeutic Sciences, University of California San Francisco, San Francisco, CA, USA; 5 IBER de Enfermedades Respiratorias, Instituto de Salud Carlos III, Madrid, Spain; 6 Center for Health Policy and Health Services Research, Henry Ford Health System, Detroit, MI, USA; 7 Department of Public Health Sciences, Henry Ford Health System, Detroit, MI, USA; 8 Departments of Medicine and Epidemiology, Johns Hopkins University, Baltimore, MD, USA; 9 Center for Genomics, Wake Forest University School of Medicine, Winston Salem, NC, USA; 10 Arizona Respiratory Center and BIO5 Institute, University of Arizona, Tucson, AZ, USA; 11 International Agency for Research on Cancer, Lyon, France; 12 Hospital Infantil Federico Gómez, México City, Mexico; 13 Department of Preventive Medicine, University of Southern California, Los Angeles, CA, USA; 14 Department of Human Genetics, University of Chicago, Chicago, IL, USA; 15 Department of Internal Medicine, Henry Ford Health System, Detroit, MI, USA; 16 Division of Intramural Research, Department of Health and Human Services, National Institute of Environmental Health Sciences, National Institutes of Health, Research Triangle, Park, NC, USA; 17 Medical Bioinformatics, Baker IDI, Melbourne, Australia

**Keywords:** Genome-wide association study, Allergic rhinitis, Coexpression network, Expression single-nucleotide polymorphism, Coexpression module, Pathway, Mitochondria, Hay fever, Allergy

## Abstract

**Background:**

Allergic rhinitis is a common disease whose genetic basis is incompletely explained. We report an integrated genomic analysis of allergic rhinitis.

**Methods:**

We performed genome wide association studies (GWAS) of allergic rhinitis in 5633 ethnically diverse North American subjects. Next, we profiled gene expression in disease-relevant tissue (peripheral blood CD4+ lymphocytes) collected from subjects who had been genotyped. We then integrated the GWAS and gene expression data using expression single nucleotide (eSNP), coexpression network, and pathway approaches to identify the biologic relevance of our GWAS.

**Results:**

GWAS revealed ethnicity-specific findings, with 4 genome-wide significant loci among Latinos and 1 genome-wide significant locus in the GWAS meta-analysis across ethnic groups. To identify biologic context for these results, we constructed a coexpression network to define modules of genes with similar patterns of CD4+ gene expression (coexpression modules) that could serve as constructs of broader gene expression. 6 of the 22 GWAS loci with P-value ≤ 1x10^−6^ tagged one particular coexpression module (4.0-fold enrichment, P-value 0.0029), and this module also had the greatest enrichment (3.4-fold enrichment, P-value 2.6 × 10^−24^) for allergic rhinitis-associated eSNPs (genetic variants associated with both gene expression and allergic rhinitis). The integrated GWAS, coexpression network, and eSNP results therefore supported this coexpression module as an allergic rhinitis module. Pathway analysis revealed that the module was enriched for mitochondrial pathways (8.6-fold enrichment, P-value 4.5 × 10^−72^).

**Conclusions:**

Our results highlight mitochondrial pathways as a target for further investigation of allergic rhinitis mechanism and treatment. Our integrated approach can be applied to provide biologic context for GWAS of other diseases.

## Background

Allergic rhinitis is an IgE-mediated inflammation of the upper airway that causes naso-ocular congestion, pruritis, rhinorrhea, and sneezing [1]. Colloquially referred to as hay fever, seasonal allergies, and allergies, allergic rhinitis is one of the most common chronic diseases, affecting up to 30% of adults and 40% of children [1].

A genetic contribution to allergic rhinitis is evident, based on an increased incidence and prevalence of allergic rhinitis among twins and within atopic families [2,3]. Despite the high population prevalence of allergic rhinitis, there have been relatively few studies of its genetic basis. The National Human Genome Research Institute catalogs just one genome wide association study (GWAS) of allergic rhinitis [4], for example, compared to 33 for asthma and 61 for diabetes [5]. Candidate gene studies have been performed with variable effect sizes and levels of significance reported [3,6,7]. We are aware of three prior GWAS of allergic rhinitis. Andiappan et al. found no genome-wide significant loci in a GWAS of allergic rhinitis in 942 Chinese subjects [8]. Ramasamy et al. reported one genome-wide significant locus in a GWAS meta-analysis of 12,898 Europeans [4]. Hinds et al. reported 16 genome-wide significant loci for self-reported allergy in a GWAS meta-analysis of subjects of European ancestry [9]. The functional implications of the identified loci were not directly examined in these studies. In a GWAS of allergen-specific IgE level (i.e. not allergic rhinitis), Bonnelykke et al. estimated that ten loci associated with allergen-specific IgE level accounted for 25% population-attributable risk for allergic rhinitis [10], but this was not from a direct study of allergic rhinitis. Of note, loci associated with allergen-specific IgE level have not been consistently associated with allergic rhinitis [4,9]. Given that the genetic loci identified to date do not fully explain the estimated heritability of allergic rhinitis, it is likely that as yet unidentified genes and pathways contribute to allergic rhinitis pathogenesis.

GWAS results on their own, while helping to elucidate the etiology of disease, do not provide a rich context within which to interpret any finding [11,12]. For example, for disease-associated SNPs in intergenic regions, the gene is not necessarily immediately known [13]. Typically the closest gene is identified as the gene of interest, but that is not a foolproof algorithm, and the pathways affected by the genetic locus are also not necessarily immediately apparent [13]. In addition, given the stringent P value thresholds that must be adopted in a GWAS to declare genome-wide significance, much of the data in a GWAS that may inform on disease is ignored because the association P values (and effect sizes) that reflect true associations cannot be distinguished from the noise [14].

Various methods have been tried to identify biologic context for loci identified by GWAS, including (1) expression quantitative trait loci (eQTL) mapping and expression single nucleotide polymorphism (eSNP) analysis [10,15-18], (2) network analysis [19,20], and (3) pathway analysis [18,21-23]. eQTL mapping and eSNP analysis are frequently used [15-18]. The motivation for eQTL mapping and eSNP analysis is that genetic variation is more likely to impact a disease trait if it alters gene transcription. Linkage or association methods can be used to identify genetic loci influencing gene expression. The linkage-based identification of loci for gene expression is called eQTL mapping, and the association-based identification of SNPs affecting gene expression is called eSNP analysis [15]. Because complex traits such as allergic rhinitis are unlikely to be governed by single genes or loci, however, eQTL and eSNP analyses alone may provide insufficient context. Network approaches can model vast networks of gene interactions that modulate disease [19,20,24]. Networks are formed by considering pairwise relationships between genes, including protein interaction relationships and coexpression relationships [14,24]. Considering GWAS results in the context of whole-gene networks may thus provide the necessary context within which to interpret the disease role for a given gene or variant identified by GWAS. Finally, pathway analysis can help decipher the functional implications of coherent groups of genes with respect to gene ontology functional categories [18,21-23]. Pathways representing specific biologic mechanisms may be overrepresented in genes identified by GWAS, thereby providing relevant biologic context for GWAS results.

Among all GWAS, some have reported findings without characterizing the effects of loci on gene expression and downstream biologic pathways [4,8,25], while others have incorporated eQTL/eSNP, network analysis, and pathway analysis individually to provide some evidence for downstream effect [15-17,19,21,23]. Integrative approaches have elucidated biologic mechanisms and treatment targets in a number of disease areas including inflammatory bowel disease, Alzheimer’s disease, diabetes, heart disease, and obesity [15,24,26-29], but similar strategies have not been widely applied to allergy. Further, the gene expression data used to support many GWAS are drawn from individuals distinct from those who were genotyped [16,18,19,21,23], rendering the analysis of any effects of genotype on gene expression indirect and potentially biased due to differences in subjects who were genotyped versus subjects with mRNA data. For example, while Hinds et al. performed GWAS to identify allergy-related loci in a sample of personal genetics company customers and birth cohort participants [9], they then identified expression quantitative trait loci (eQTL) among these loci using monocyte gene expression data from a distinct study cohort of heart disease.

We hypothesized that a genome-wide approach to allergic rhinitis integrating GWAS with eSNP, coexpression, and pathway analyses using gene expression data generated from disease-relevant tissue collected from the same individuals who were genotyped could enhance the power over standard GWAS to identify disease-relevant loci. Such an approach could not only provide more robust biological context, but also leverage data from cohorts that may not be large enough to yield high numbers of genome-wide significant GWAS results for complex traits such as allergic rhinitis. Here we present our integrated genomic analysis of allergic rhinitis, where we not only identified genome-wide significant genetic variants associated with allergic rhinitis, but also explored the biologic context for these results by profiling gene expression from CD4+ lymphocytes collected from genotyped subjects and performing expression single nucleotide polymorphism (eSNP), network, and pathway analyses. Our integrated approach identified a novel pathway in allergic rhinitis.

## Results

**Figure 1:**
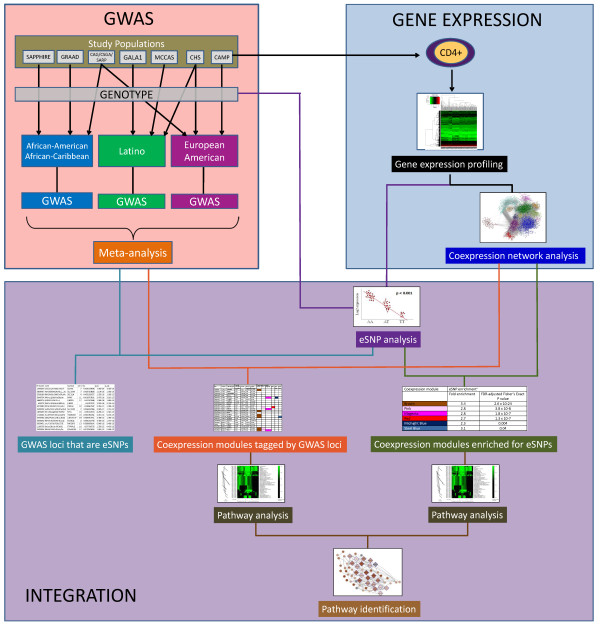
**Study flow for the integrated genome-wide association, coexpression network, and expression single nucleotide polymorphism analysis of allergic rhinitis.** CHS = Children’s Health Study, CAMP = Childhood Asthma Management Program, CAG = Chicago Asthma Genetics Study, CSGA = Collaborative Studies on the Genetics of Asthma, SARP = Severe Asthma Research Program, GALA1 = Genetics of Asthma in Latinos, MCCAS = Mexico City Childhood Asthma Study, GRAAD = Genomic Research on Asthma in the African Diaspora and Barbados, SAPPHIRE = Study of Asthma Phenotypes and Pharmacogenomic Interactions by Race-Ethnicity. Detailed descriptions of the individual studies have been previously described [25].

Our integrated genomic analysis of allergic rhinitis yielded results from GWAS, gene expression profiling, and their integrated analysis (Figure 1). We first describe the results of our GWAS of allergic rhinitis in 5633 ethnically diverse North American subjects, where we identified genome-wide significant loci that were specific to ethnicity (Figure 1, pink box). We then describe the results of our gene expression profiling of immune cells key to allergy (CD4+ lymphocytes [30]), collected from the peripheral blood of selected subjects who had undergone GWAS (Figure 1, blue box). We share the results for the weighted gene coexpression network [31] we constructed to identify modules of genes expressed together. Finally, we describe the integration of our GWAS and gene expression analyses (Figure 1, purple box), where we performed eSNP analysis to assess for the association between genetic variation and gene expression (Figure 1, purple path), assessed GWAS loci for eSNPs (Figure 1, turquoise path), identified coexpression modules tagged by GWAS loci (Figure 1, orange path), and analyzed coexpression modules for enrichment of allergic-rhinitis associated eSNPs (Figure 1, green path) [15]. We then used pathway analysis to further inform on the biological context for our integrated findings.

### GWAS

#### Subject characteristics

The baseline characteristics of the participating subjects are shown in Table 1. In total, there were 5633 subjects from 7 EVE Consortium study centers [25] in the United States, Mexico, and Barbados who were assessed for allergic rhinitis. 2756 (49%) were female. Participants were diverse, with 2034 (36%) European American, 2326 (41%) Latino, and 1273 (23%) African American/African Caribbean. The overall prevalence of allergic rhinitis cases was 48% (2712 subjects).

**Table 1:** Baseline characteristics of North American subjects included in the study

	**Study**^**a**^						
	**CHS**	**CAMP**	**CAG/CSGA/SARP**	**GALA1**	**MCCAS**	**GRAAD**	**SAPPHIRE**
Number	2881	384	283	521	476	809	279
Age (years)	8.3 (5.2-14.3)	8.8 (5.2-13.2)	27.3 (6.0-81.0)	14.8 (8.0-40.0)	9.0 (5.0-17.0)	40.0 (14.0-84.0)	30.3 (12.0-56.0)
Female	1344 (47%)	142 (37%)	150 (53%)	230 (44%)	198 (42%)	474 (59%)	219 (78%)
Race							
European American	1552 (54%)	384 (100%)	98 (35%)				
Latino	1329 (46%)			521 (100%)	476 (100%)		
African American/African Caribbean			185 (65%)			809 (100%)	279 (100%)
Allergic Rhinitis	1096 (38%)	199 (52%)	245 (87%)	434 (83%)	250 (53%)	377 (47%)	111 (40%)
Asthma	1206 (42%)	384 (100%)	283 (100%)	521 (100%)	476 (100%)	228 (28%)	148 (53%)
Genotyping platform^b^	550 K, 610 K	550 K	1Mv1	6.0	550 K	650 K	6.0

Values are mean (range) or number (percent).

^a^CHS = Children’s Health Study, CAMP = Childhood Asthma Management Program, CAG = Chicago Asthma Genetics Study, CSGA = Collaborative Studies on the Genetics of Asthma, SARP = Severe Asthma Research Program, GALA1 = Genetics of Asthma in Latinos, MCCAS = Mexico City Childhood Asthma Study, GRAAD = Genomic Research on Asthma in the African Diaspora and Barbados, SAPPHIRE = Study of Asthma Phenotypes and Pharmacogenomic Interactions by Race-Ethnicity. Detailed descriptions of the individual studies have been previously described [25].

^b^The Illumina arrays used were the 1Mv1, 550 k, 610 k and 650 k. The Affymetrix arrays used were the 500 k and 6.0.

#### GWAS and meta-analysis

**Figure 2:**
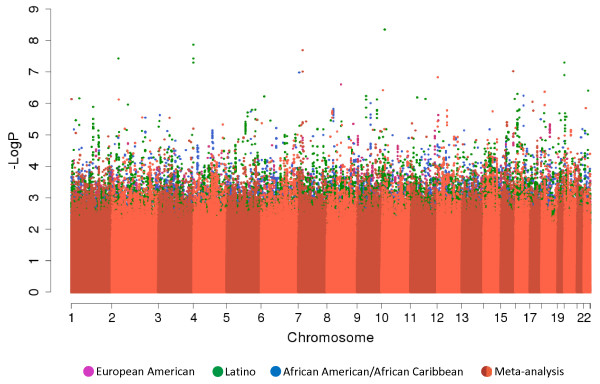
Manhattan plot of the genome-wide association and meta-analysis results for allergic rhinitis showing ethnicity-specific findings.

**Figure 3:**
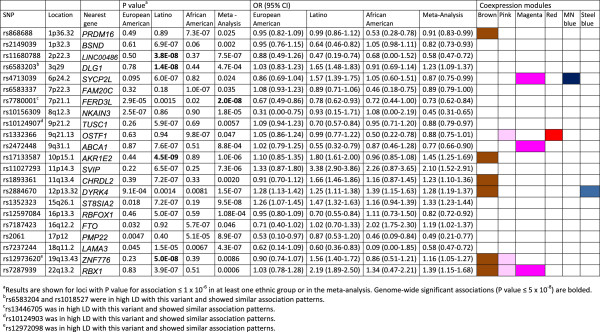
Results of the genome-wide association studies of allergic rhinitis, meta-analysis, and GWAS tagging of the coexpression network.

Because subjects were ethnically diverse, we pooled genotype data from the 7 study centers into three ethnic groups for GWAS: European American, Latino, and African-American/African Caribbean (Figure 1, pink box) and controlled for population stratification within each ethnic group using principal components. Figure 2 shows the results of genome-wide association studies for allergic rhinitis among European Americans, Latinos, and African-American/African-Caribbeans, in addition to the results of the meta-analysis across these ethnic groups. For additional views, Additional file 1: Figure S1 shows the Manhattan plots separately for each ethnic group and for the meta-analysis. There were distinct findings for each ethnic group. Figure 3 summarizes the results for the 22 loci with P value for association ≤ 1 × 10^−6^ in at least one of the ethnic groups or in the meta-analysis. We show loci meeting this threshold to include loci with suggestive associations (P value ≤ 1 × 10^−6^) in addition to those genome-wide significant (defined as P value ≤ 5 × 10^−8^), as loci not meeting strict definitions of genome-wide significance can have biologic relevance [11,12]. Allele frequencies are shown in Additional file 2: Table S1, and a QQ plot for the GWAS meta-analysis is shown in Additional file 3: Figure S2. The genomic inflation factor was 1.06, supporting adequate control for population stratification.

Four loci on chromosomes 2p22.3 near *LINC0048*, 3q29 near *DLG1*, 10p15.1 near *AKR1E2*, and 19q13.43 near *ZNF776* were genome-wide significant among Latinos (Figure 3). The regional association plots for these loci are shown in Additional file 4: Figure S3. For context, each of these SNPs was directly genotyped in 2 of the 7 populations, imputation was performed using very conservative metrics [25], and the imputation scores for these SNPs demonstrated good confidence (Additional file 5: Table S2). The regional LD plots for these loci (Additional file 6: Figure S4) show that there were limited SNPs in LD with these genome-wide significant loci. The locus marked by rs7780001 on chromosome 7p21.1 near *FERD3L* was genome-wide significant in the meta-analysis across ethnic groups (P value 2.0 × 10^−8^; Figure 3 and Additional file 7: Figure S5) and had nominally significant associations in all three ethnic groups. The loci marked by rs2884670 on chromosome 12p13.32 near *DYRK4* and rs7237244 on chromosome 18q11.2 near *LAMA3* also had nominally significant associations in all three ethnic groups. Among the 17 loci previously identified by GWAS as associated with allergic rhinitis [4,9], four were associated with allergic rhinitis with P value ≤ 0.05 in our study (Additional file 8: Table S3).

Individuals with allergic rhinitis frequently have comorbid asthma [1,32]. Indeed, we observed that 2051 (76%) of those with allergic rhinitis had asthma, and 1195 (41%) of those without allergic rhinitis had asthma. As subphenotypes of AR based on asthma status are possible, we also performed secondary GWAS stratified by asthma status. These results are shown in the supplementary file (Additional file 9: Supplementary Results 1, Additional file 10: Table S4**,** and Additional file 11: Figure S6) and similarly showed ethnicity-specific findings. In Additional file 12: Table S5, we show the sample composition of the stratified analyses according to asthma status.

### Genome-wide CD4+ gene expression and coexpression network to enhance GWAS

To assess the potential biological impact of the loci identified in our GWAS analyses, we collected and measured genome-wide gene expression in disease-relevant tissue (peripheral blood CD4+ lymphocytes) from 200 subjects who had undergone GWAS and constructed a gene coexpression network based on the gene expression data (Figure 1**,** blue box). We built the coexpression network to identify coexpressed gene modules (i.e. groups of genes with similar patterns of expression profiles and interconnectivity across the experimental samples), as these could serve as broader constructs of gene expression and provide a path to discover broader biologic context [31].

**Figure 4:**
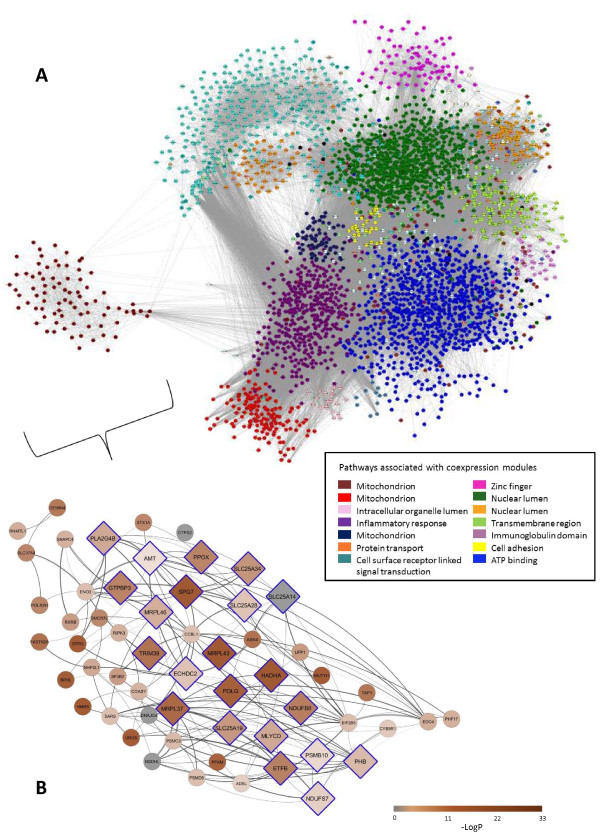
**CD4+ lymphocyte coexpression network with detail of the brown coexpression module. A**. Each circle represents a gene. Weighted gene coexpression analysis identified groups of genes with similar patterns of gene expression and interconnectivity (coexpression modules). The 41 coexpression modules identified are labeled by color. Pathways associated with the largest coexpression modules are denoted in the legend. **B**. Interconnectivity of the brown coexpression module is shown in detail. Tagged by 6 allergic rhinitis GWAS loci, this coexpression module was highly enriched for allergic rhinitis-associated eSNPs (3.4-fold enrichment, FDR-adjusted P value = 2.6 × 10^−24^) and also highly enriched for pathways related to mitochondrial function (8.6-fold enrichment, FDR-adjusted P value = 4.5 × 10^−72^). Genes containing allergic rhinitis-associated eSNPs are marked in brown, with those containing eSNPs with lowest P-value for association between genotype and gene expression marked with greatest brown saturation. Genes in pathways related to mitochondrial function are marked by diamonds with blue outline. Higher correlation between gene expression is shown with thicker and darker edges.

We achieved CD4+ lymphocyte yields of ~4x10^6^ cells at ≥95% purity per collection. Bioanalyzer (Agilent Technologies, Santa Clara, CA) analysis confirmed average total RNA yields of 2 μg per collection, with minimal evidence of RNA degradation and 28S:18S ratios approaching 2.0.Figure 4A shows the coexpression network we constructed using weighted gene coexpression network analysis of the CD4+ lymphocyte gene expression data. In total, there were 41 coexpressed gene modules identified by the coexpression network, and their interconnectivities are shown. For ease of visualization, modules are identified by color.Using pathway analysis, we found that the modules were enriched for a variety of gene ontology (GO) pathways reflecting the functions being carried out by each module. Pathways associated with the largest coexpression modules are shown in the legend of Figure 4A. For example, the brown module highlighted in Figure 4B was enriched for mitochondrial function. Zinc finger, inflammatory response, and immunoglobulin domain were other pathways highlighted by examining the coexpression modules for functional enrichment (Figure 4A).

### Integration of GWAS and CD4+ gene expression to explore biologic context for GWAS

To explore the biologic context for our GWAS results, we analyzed our GWAS and gene expression findings together (Figure 1, purple box).

#### GWAS loci that are eSNPs

We first performed eSNP analysis to assess for the association between genetic variation and gene expression (Figure 1, purple path). We then examined the GWAS and eSNP results together to identify GWAS loci that were eSNPs (Figure 1, turquoise path), as genetic variation that is associated with both the trait and gene expression is more likely to be biologically relevant than variants that are associated with the trait only with no effect on gene expression. We found that the 19q13.43 locus near *ZNF776* was associated with allergic rhinitis (GWAS P value 5.0 × 10^−8^) as well as CD4+ gene expression (*χ*^2^ = 19.55, FDR-adjusted P value 0.00078). The other loci identified by GWAS were not associated with CD4+ gene expression.

Given the relatively modest size of our sample and the fact that we were examining a complex trait, we had anticipated that traditional GWAS would uncover only a small number of biologically relevant loci, even with the aid of eSNP analysis, as such an approach would rely upon detection of single variant associations with trait and expression. We therefore sought to leverage the CD4+ expression data more broadly through coexpression network and pathway analysis.

#### Coexpression modules tagged by GWAS loci

Compared to individual genes, coexpression modules identified through coexpression network analysis (Figure 4) can serve as more general constructs of gene expression, providing a path to discover broader context and related loci [14,28,31,33-40]. Motivated by the same rationale that genetic variation that is associated with both the trait and gene expression is more likely to be biologically relevant than variants associated with the trait only, we mapped GWAS loci to CD4+ coexpression modules and examined the modules that were tagged by GWAS loci (Figure 1, orange path). Specifically, we defined a GWAS locus as tagging a coexpression module if a coexpression module contained a gene within 250 kb of the locus. We found that 9 of the 22 GWAS loci tagged at least one coexpression module and 6 coexpression modules in total (Figure 3). These 6 modules (the brown, pink, magenta, red, midnight blue, and steel blue coexpression modules) tagged by GWAS loci were therefore considered candidate allergic rhinitis associated modules that could inform on allergic rhinitis biology. The 19q13.43 locus near *ZNF776* was among the GWAS loci tagging coexpression modules, corroborating our eSNP results and illustrating the increased power of detection gained by using the coexpression module as a more general construct of gene expression. Of note, some of candidate allergic rhinitis associated modules were tagged by GWAS loci that would not have been considered remarkable by traditional criteria for genome-wide significance of individual loci (P value ≤ 5.0 × 10^−8^). Our approach of using GWAS loci to tag coexpression modules therefore allowed us to gain additional utility from our GWAS results.Among the 6 candidate allergic rhinitis associated modules tagged by GWAS loci, the brown module (representing mitochondrial pathways according to pathway analysis (Figure 4)) was tagged by 6 of the 22 GWAS loci (Figure 3). This proportion represented a significant enrichment (4.0-fold enrichment, P-value 0.0029) over chance, supporting a connection between these GWAS loci and mitochondrial pathway functions.

#### Coexpression module enrichment for allergic rhinitis-associated eSNPs

To further ascertain whether any of the 6 candidate allergic rhinitis associated modules were underlying allergic rhinitis susceptibility, we tested whether these modules were enriched for eSNPs that were also associated with allergic rhinitis (Figure 1, green path). eSNPs (i.e. SNPs associated with gene expression) represent functionally validated SNPs of interest in that they are associated with expression levels of genes in a cell type relevant to the disease under study [15]. While such individual associations may not be meaningful, the pattern of associations enriched within a given coexpression module can provide strong statistical support for module involvement at the genetic level in the disease [14,28,31,33-40]. The additional association of eSNPs within a coexpression module with the disease of interest provides further statistical support that the coexpression module is involved in the disease, as the module is then not only enriched for eSNPs (e.g. SNPs associated with CD4+ gene expression), but more specifically, enriched for disease-associated eSNPs (e.g. SNPs associated with both CD4+ gene expression and allergic rhinitis).

**Figure 5:**
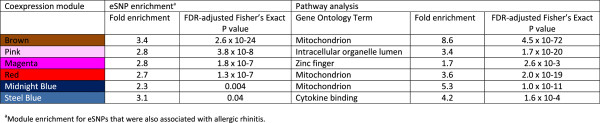
eSNP enrichment and pathway analysis of coexpression modules tagged by GWAS loci.

In this instance, we identified the brown module as giving rise to the greatest enrichments of allergic-rhinitis-associated eSNPs (3.4-fold enrichment; FDR-adjusted Fisher’s Exact Test P value 2.6 × 10^−24^) (Figure 5), thus providing statistical support for involvement of the brown module in allergic rhinitis. Pathway analysis revealed that the brown module was enriched for mitochondrial pathways (8.6-fold enrichment, FDR-adjusted Fisher Exact Test P value = 4.5 × 10^−72^) (Figure 5 and Figure 4B). The red and midnight blue modules were also enriched for allergic rhinitis–associated eSNPs, and both these modules were enriched for mitochondrial pathways as well (Figure 5). The pink module, enriched for the GO term intracellular organelle lumen, functionally overlaps with mitochondrial pathway. Thus, the candidate allergic rhinitis-associated modules were all significantly enriched for allergic rhinitis-associated eSNPs, and pathway analysis results for at least half of these modules highlighted mitochondrial pathways as linked to allergic rhinitis. Results from randomized networks did not yield meaningful results (Additional file 13: Supplementary Results 2, Additional file 14: Figure S7).

In summary, pathway analysis of our results from the integration of GWAS and coexpression network analysis (showing a significantly high number of GWAS loci tagging the brown module), as well as from the integration of eSNP analysis and coexpression network results (showing greatest enrichment of the brown module for allergic rhinitis-associated eSNPs), both pointed to mitochondrial pathways as playing an important role in allergic rhinitis (Figure 1). Key to these results was that the coexpression network helped organize the expression traits into coherent, highly interconnected modules reflecting the biological processes at play in the tissue. By using GWAS loci to tag coexpression modules and then requiring these tagged coexpression modules to be enriched for eSNPs that were also associated with allergic rhinitis, we were able to place candidate GWAS associations in a more informed context that not only provided a biological context for GWAS interpretation, but enhanced confidence in the suggestive hits given an enrichment of multiple functional SNPs associating with the disease phenotype [15]. Through this approach, we found consistent evidence that mitochondrial pathways likely play a role in the genetics and pathophysiology of allergic rhinitis.

## Discussion

A motivation for genome-wide studies is the desire to identify novel pathways and mechanisms in disease pathogenesis. A limitation of traditional genome-wide association studies is that statistically significant loci may be identified [4,9], but the biological relevance of the individual or aggregate variants are often not evident [11,12]. This is not overcome by replication of genotype associations, which has been the usual path taken to follow up GWAS findings, and one which has led to limited success [7,9,41]. Our study demonstrates the advantages of integrating network approaches with GWAS to identify and prioritize pathways and gene targets of biologic relevance. By integrating our GWAS findings with eSNP, coexpression, and pathway analyses using gene expression data from disease-relevant tissue generated from subjects who had undergone GWAS, we tested the potential biologic context of our GWAS findings through integrative methods and identified a novel pathway in allergic rhinitis—mitochondrial pathways. Our method allowed us to leverage data from multiple GWAS loci to identify biologic context for the aggregate findings. This is in contrast to traditional GWAS, where the implications of individual SNP associations are often challenging to define [11,12]. Because complex traits such as allergic rhinitis are unlikely to be governed by single variants, strategies that capitalize on broader constructs of GWAS and gene expression results are more likely to yield informative disease context. We adopted such a strategy and were able to identify novel biologic context for allergic rhinitis. Further, our approach of integrating genotype and gene expression data generated from the same sample has not been widely applied to the study of allergic diseases. Our methods can be used to provide a richer biologic context for GWAS findings in other disease areas.

While mitochondrial pathways have not been associated with allergic rhinitis pathogenesis in traditional descriptions [1] or genetic studies of the disease [4,8,9], our findings and those from laboratory-based studies of airway dysfunction support a role for mitochondrial perturbations in allergic rhinitis pathogenesis. There is a strong link between upper (e.g. nasal) and lower (e.g. bronchial) airway disease pathogenesis [42], and mitochondrial perturbations have been observed to affect airway inflammation. Mitochondria are the major source of endogenous reactive oxygen species, which are required for normal function of the acquired immune response, including normal T-cell activation, B-cell differentiation, and T-cell and B-cell proliferation [43]. Because alterations in the acquired immune response are observed in allergic inflammation and allergic rhinitis, mitochondrial disruption could play a role in allergic rhinitis. There are some experimental data in support of this hypothesis. OVA-induced allergic airway inflammation in BALB/c mice triggers mitochondrial dysfunction, including the reduction of cytochrome c oxidase activity in lung mitochondria, reduction in the expression of subunit III of cytochrome c oxidase in bronchial epithelium, appearance of cytochrome c in lung cytosol, and mitochondrial ultrastructural changes such as loss of cristae and swelling [44]. Experiments using pollen, rather than ova, to induce allergic inflammation more akin to allergic rhinitis in humans, have also shown mitochondrial disturbance. Pollen grains and subpollen particles have intrinsic NADPH oxidases [45]. Upon hydration in the airway epithelium they produce reactive oxygen species that induce oxidative stress [46]. The pollen-induced oxidative stress damages mitochondrial respiratory chain proteins (specifically NADH dehydrogenase Fe-S protein (NDUFS) and ubiquinol-cytochrome c reductase core (UQCRC)) in human airway epithelial cells, triggers reactive oxygen species production from mitochondrial respiratory chain complex III, and induces mitochondrial dysfunction in complex III [47].

Mitochondrial changes induced by pollen can serve as the second hit leading to allergic inflammation if there is preexisting mitochondrial dysfunction. Indeed, when treated intranasally with pollen extract, mice with mitochondrial dysfunction (induced deficiency in UQCRC2) demonstrated evidence of allergic airway inflammation, in contrast to UQCRC2-sufficient control mice challenged with the same pollen extract [47]. Specifically, these mice with mitochondrial dysfunction exhibited a 4.4 fold increase in bronchoalveolar lavage eosinophil counts, increased accumulation of peribronchial inflammatory cells, enhanced mucous cell metaplasia in airway epithelium, and increased airway hyperresponsiveness [47]. Although these studies characterized changes in the lower airway, the strong link between upper and lower airway disease pathogenesis [42] suggests that analogous changes in the upper airway could cause individuals with preexisting mitochondrial dysfunction to develop allergic inflammation leading to allergic rhinitis with pollen exposure. Our results highlight this as a potential mechanistic area, as 27% (6/22) of the genetic loci for allergic rhinitis that we identified by genome-wide association analysis tagged a gene coexpression module that was not only markedly enriched for eSNPs associated with allergic rhinitis (3.4-fold, FDR-adjusted P-value 2.6 × 10^−24^), but also significantly enriched for mitochondrial pathways by pathway analysis (8.6-fold enrichment, FDR-adjusted P value 4.5 × 10^−72^).

Population-based studies additionally support a role for mitochondrial pathways in allergic rhinitis pathogenesis. Mitochondria are the primary sites of oxidative reactions. Levels of malondialdehyde (a marker of oxidative stress) are higher, and levels of reduced glutathione (an antioxidant) are lower in the exhaled nasal condensates of allergic rhinitis subjects compared to healthy controls [48]. The epidemiological link between maternal history of atopy (as opposed to paternal history of atopy) and greater risk for allergic rhinitis in offspring [49,50] may be explained by the fact that mitochondria are maternally transmitted. Consistent with this, mitochondrial haplotypes are associated with intermediate phenotypes of allergic rhinitis, including total serum IgE levels and skin prick test reactivity [51].

Our results suggest that reducing mitochondrial dysfunction could improve allergic rhinitis. In murine models of allergic airway inflammation, intratracheal administration of an antioxidant known to enter mitochondria and protect the electron chain from oxidative damage [52] decreased allergen-induced airway hyperreactivity and airway inflammation severity, as shown by reduced numbers of inflammatory cells in bronchoalveolar fluid [53]. Again, these studies focused on lower airway disease, but the strong link between upper and lower airway disease pathogenesis [42] suggests that it may be possible to achieve similar results if the upper airway were targeted in allergic rhinitis treatment.

Our GWAS of allergic rhinitis was the first to examine ethnically diverse subjects for this complex trait, and our study revealed susceptibility loci that were specific to ethnicity. This is consistent with genome-wide association studies of other complex diseases-- such as asthma [25,54] and obesity [55,56]-- that have also demonstrated ethnicity-specific effects. Given the possibility that allergic rhinitis with comorbid asthma vs. allergic rhinitis without comorbid asthma may be distinct disease subphenotypes [32], we also performed secondary GWAS analyses stratified by asthma status. These results similarly showed ethnicity specific findings. Our findings support the utility of studying ancestrally-diverse populations in genome-wide studies.

We recognize the limitations of our study. We defined allergic rhinitis using criteria commonly employed in population-based and genetic studies of allergic rhinitis, which are based on questionnaire without objective markers [1,4,9]. For our eSNP and coexpression network analyses, it would have been ideal to have profiled gene expression for all 5633 subjects who participated in the GWAS, but CD4+ lymphocytes were not available from all subjects. We had CD4+ lymphocytes from European-American CAMP subjects only, and it is possible that coexpression results would have differed had we additionally had expression profiles from subjects of other ethnic backgrounds, as gene expression can vary by ethnicity. While expression differences can change with ethnicity, the connectivity structure is expected to be much more highly conserved, however, and is even seen across species [57]. Additionally, we recognize that our coexpression network may have yielded distinct results had we chosen a different tissue for gene expression profiling; we had chosen to study peripheral blood CD4+ lymphocytes given their central role in allergic disease [30]. Despite these limitations, we were able to implement an integrative analysis of our GWAS, coexpression network, and eSNP results, leading to the identification of a novel biologic pathway in allergic rhinitis. Our strategy created an informed biological context for our GWAS that may be used to better understand allergic rhinitis. Further, our methods may be implemented to provide biologic context for GWAS of other diseases.

## Conclusions

Our GWAS of allergic rhinitis of 5633 ethnically diverse subjects demonstrated ethnicity-specific, genome-wide significant findings. To determine the potential biological impact of the variants identified in our GWAS, we integrated eSNP, coexpression network, and pathway analyses using gene expression data generated from subjects who had undergone GWAS. Our integrated approach identified mitochondrial pathways as important in allergic rhinitis, and our strategy may prove useful to studying other diseases.

## Methods

### Ethics statement

Each study was approved by the Institutional Review Board of the corresponding institution. Informed consent was obtained for all study participants, and where appropriate, informed assent from minors and informed consent from their parent were obtained.

### Subjects, genotyping, and phenotyping

Subjects were recruited from EVE Consortium centers in the United States, Mexico, and Barbados. Detailed descriptions of the individual studies, genotyping platforms, and quality control protocols have been previously described [25]. Of note, SNPs with imputation quality scores below a threshold (Rsq < 0.3) were removed from the analysis. We included subjects who were specifically assessed for allergic rhinitis, and these came from 7 study centers (Figure 1). Allergic rhinitis status was considered positive if a subject reported a history of allergic rhinitis ever, defined as hay fever or runny/stuffy nose with sneezing or itching when the subject did not also have a cold or flu.

### GWAS and meta-analysis

Summary files on a common set of SNPs were shared among the EVE Consortium investigators. Genotype imputation using HapMap reference panels and Markov Chain Haplotyping software (MaCH) [58] were performed in each sample as previously described [25]. We pooled the imputed genotype data for each ethnic group (European American, Latino, African American/African Caribbean) (Figure 1). To adjust for potential population stratification, we used Eigenstrat [59] to create principal components for each ethnic group. Within each ethnic group, we tested for the association of SNPs with allergic rhinitis by constructing a test statistic that had a standard normal distribution under the null hypothesis of no association and captured the direction of the effect. Models were implemented in PLINK [60] and controlled for age, sex, and principal components. To allow for comparability with previous GWAS of allergic rhinitis [4,8,9], we did not include asthma status as a covariate. To assess for the effects of SNPs across ethnic groups, we then calculated a meta-analysis statistic as a combination of the individual ethnic study scores using METAL [61].

Recognizing the potential subphenotypes of isolated allergic rhinitis vs. allergic rhinitis with comorbid asthma [1,32], we additionally performed secondary GWAS and meta-analyses in subjects stratified by asthma status using methods analogous to the above.

### Genome-wide CD4+ gene expression

We collected peripheral blood CD4+ lymphocytes from 200 subjects who had undergone GWAS. These 200 subjects were from Childhood Asthma Management Cohort (CAMP) cohort [62], one of the member centers of the EVE Consortium (Figure 1). We focused on this sample subset because of biospecimen availability. Peripheral blood was collected into BD Vacutainer CPT tubes (BD Diagnostics, Franklin Lakes, New Jersey) and placed on ice. Samples were centrifuged within 1 hour of collection for 20 minutes at 1700RCF, followed by mononuclear cell layer isolation and suspension in 10 ml of PBS. We isolated CD4+ lymphocytes using anti-CD4+ microbeads by column separation (Miltenyi Biotec, Auburn, CA) using 20 μl anti-CD4+ Micro beads per 106 total cells. To extract total RNA, we used the RNeasy Mini Protocol (QIAGEN, Valencia, CA) and stored at −80°C. We generated expression profiles with the Illumina HumanRef8 v2 BeadChip arrays (Illumina, San Diego CA). Expression data were log2 transformed and quantile normalized.

### Coexpression network analysis

We performed weighted gene coexpression network analysis to identify coexpressed gene modules [31]. We used a previously applied, well-established, well-recognized, and validated method to construct the coexpression network [14,28,31,33-40]. For module detection, we used average linkage hierarchical clustering of a topological overlap matrix based on an adjacency matrix that is comprised of power-transformed correlations between gene expression profiles [31]. To cut branches of the tree into gene modules, we used the dynamic tree cutting algorithm, which iteratively searches for stable branch sizes and chooses clusters based on the shape of each dendrogram branch [63]. This algorithm allows manipulation of several parameters controlling the resultant cluster size and cohesiveness. The modules identified from the coexpression network were then carried forward into the integrative analysis.

To provide support for the specificity of our coexpression network, we generated multiple random coexpression networks where gene assignments were randomized (Random Networks 1–3), as well as random networks where the gene expression levels were randomized (Random Networks 4–6). We carried these random networks forward into the integrated analysis as well.

### Integration of GWAS and CD4+ gene expression

We defined a GWAS locus (P value for association ≤ 1 × 10^−6^) as tagging a coexpression module if a coexpression module contained a gene within 250 kb of the locus. Coexpression modules tagged by GWAS loci were identified as candidate allergic rhinitis associated modules, and then assessed for enrichment of eSNPs that were also nominally associated with allergic rhinitis. A SNP was considered an eSNP if the SNP was located within 1 megabase of the corresponding gene, and the association between genotype and gene expression was significant at a 10% false discovery rate (FDR) (P value ≤ 1 × 10^−4^). For modules tagged by at least 1 GWAS locus, we used the Fisher’s exact test to assess whether a module was enriched for eSNPs that were also nominally associated (P value ≤ 0.01) with allergic rhinitis. The composition of modules was then assessed by pathway analysis using defined gene ontologies (GO) via the DAVID analysis tool [64,65]. Overrepresentation of canonical pathways and biological processes in modules was measured via the Fisher’s exact test. P values from this test were FDR-adjusted given the number of modules and functional categories tested. Networks were visualized using the Cytoscape network visualization tool [66].

## Abbreviations

FDR: False discovery rate; GWAS: Genome-wide association study; eQTL: Expression quantitative trait loci; eSNP: Expression single nucleotide polymorphism; GO: Gene ontology; IgE: Immunoglobulin E; LD: Linkage disequilibrium; QQ: Quantile quantile.

## Competing interests

The authors declare that they have no competing interests.

## Authors’ contributions

Conception and design of study: SB, EES, BEH, BAR, STW, Acquisition of data: SB, BEH, RL, JPZ, DGT, CSE, MPY, BP, JJY, RAM, THB, XL, PG, IR, BRN, MTS, HV, DLN, CO, FDM, ERB, DAM, WJG, FG, EGB, KCB, LKW, SJL, BAR, STW, Analysis and interpretation of data: SB, EES, BEH, JLS, WQ, RL, JPZ, AC, ML, BZ, BAR, STW, Drafting of the manuscript: SB, EES, STW, Review of the manuscript: SB, BEH, RL, JPZ, DGT, CSE, MPY, BP, JJY, RAM, THB, XL, PG, IR, BRN, MTS, HV, DLN, CO, FDM, ERB, DAM, WJG, FG, EGB, KCB, LKW, SJL, BAR, STW, Critical revision of the manuscript: SB, EES, BEH, JPZ, BZ, STW. All authors read and approved the final manuscript.

## Pre-publication history

The pre-publication history for this paper can be accessed here:

http://www.biomedcentral.com/1755-8794/7/48/prepub

## Supplementary Material

Additional file 1: Figure S1 Manhattan plots for the European American, Latino, and African-American genome-wide association and meta-analysis results for allergic rhinitis. Click here for file

Additional file 2: Table S1 Allele frequencies for loci identified in the GWAS of allergic rhinitis. Click here for file

Additional file 3: Figure S2 QQ plot for the GWAS meta-analysis of allergic rhinitis across ethnic groups. Click here for file

Additional file 4: Figure S3 Regional associations for genome-wide significant loci (P value ≤ 5 × 10^−8^) in the GWAS of allergic rhinitis among Latinos. Click here for file

Additional file 5: Table S2 Mean Rsq values for genome-wide significant loci in the GWAS of allergic rhinitis among Latinos. Click here for file

Additional file 6: Figure S4 Regional LD plots for genome-wide significant loci (P value ≤ 5 × 10–8) in the GWAS of allergic rhinitis among Latinos**.** Click here for file

Additional file 7: Figure S5 Regional associations for the genome-wide significant locus (P value 1.0 × 10^−8^) in the GWAS meta-analysis across ethnic groups. Click here for file

Additional file 8: Table S3 P values for association with allergic rhinitis for 17 loci identified in previous GWAS of allergic rhinitis. Click here for file

Additional file 9: Supplementary Results 1 Results for the GWAS of allergic rhinitis stratified by asthma status. Click here for file

Additional file 10: Table S4 Results of the genome-wide association studies of allergic rhinitis among subjects without asthma. Click here for file

Additional file 11: Figure S6 Regional associations for the locus with suggestive associations in the GWAS of allergic rhinitis among African Americans/African Caribbeans with asthma. Click here for file

Additional file 12: Table S5 Sample composition of the stratified analysis according to asthma status. Click here for file

Additional file 13: Supplementary Results 2 Random coexpression networks. Click here for file

Additional file 14: Figure S7 Plots of scale free topology for coexpression networks constructed from randomized CD4+ gene expression data. Gene names were randomized in Random Networks 1–3. Gene expression values were randomized in Random Networks 4–6. Click here for file
