# Supplementary material for: Integrated genome-wide association, coexpression network, and expression single nucleotide polymorphism analysis identifies novel pathway in allergic rhinitis
Source: BMC Med Genomics. 2014 Aug 2;7:48. doi: 10.1186/1755-8794-7-48 (PMC4127082; doi:10.1186/1755-8794-7-48)

Figure S1: Manhattan plots for the European American, Latino, and African American/African Caribbean genome-wide association and meta-analysis results for allergic rhinitis

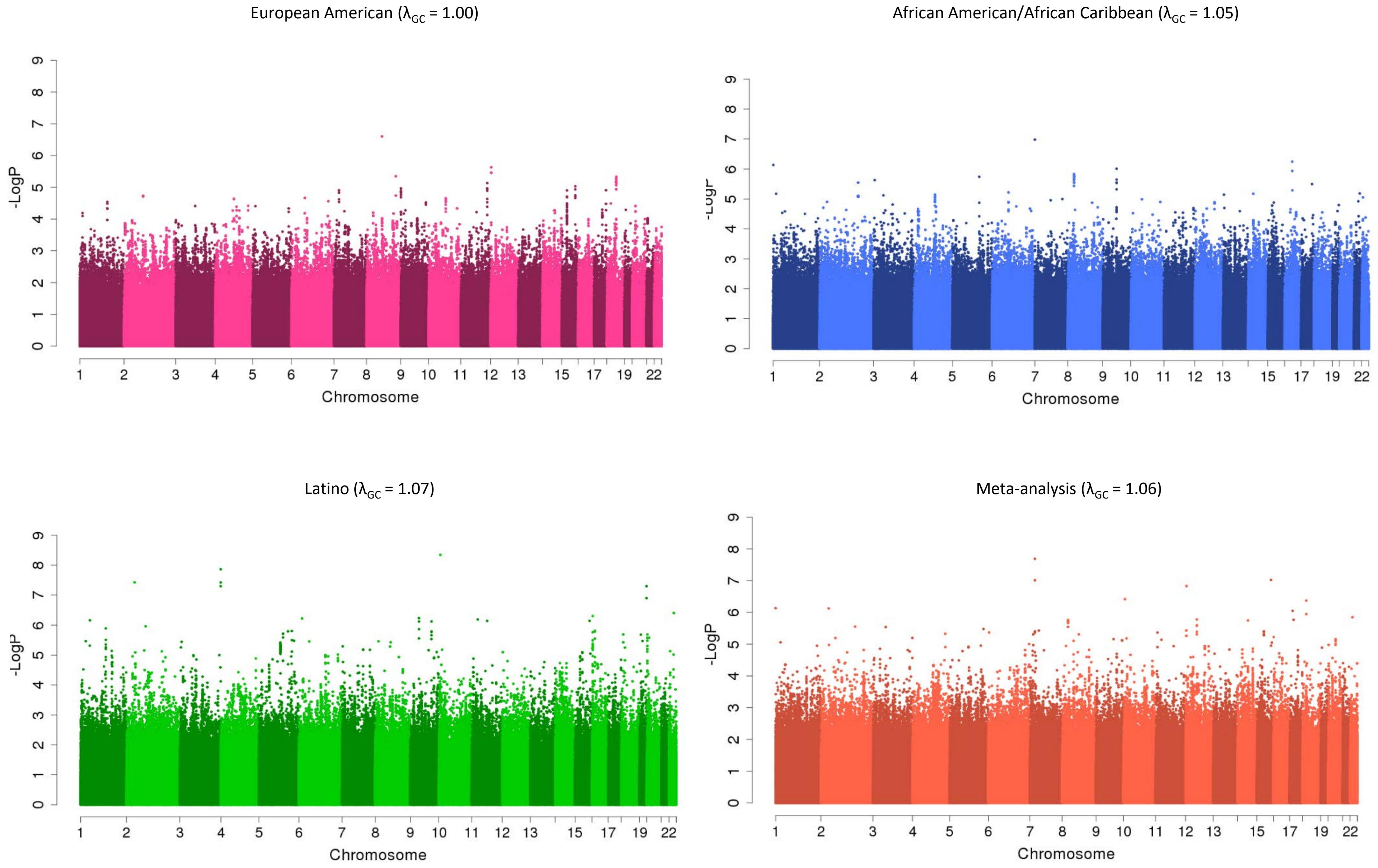

Supplement: Additional file 1: Figure S1 — Manhattan plots for the European American, Latino, and African-American genome-wide association and meta-analysis results for allergic rhinitis. [file 1755-8794-7-48-S1.pdf]
