# Supplementary material for: Integrated genome-wide association, coexpression network, and expression single nucleotide polymorphism analysis identifies novel pathway in allergic rhinitis
Source: BMC Med Genomics. 2014 Aug 2;7:48. doi: 10.1186/1755-8794-7-48 (PMC4127082; doi:10.1186/1755-8794-7-48)
Supplement: Additional file 2: Table S1 — Allele frequencies for loci identified in the GWAS of allergic rhinitis. [file 1755-8794-7-48-S2.pdf]

Table S1: Allele frequencies for loci identified in the GWAS of allergic rhinitis

SNP	Location	Allele*	European American	Latino	African American
rs868688	1p36.32	T	0.52	0.43	0.39
rs2149039	1p32.3	A	0.79	0.68	0.78
rs11680788	2p22.3	C	0.95	0.90	0.97
rs6583203	3q29	C	0.34	0.33	0.38
rs4713039	6p24.2	A	0.81	0.81	0.94
rs6583337	7p22.3	A	0.38	0.28	0.32
rs7780001	7p21.1	A	0.86	0.78	0.78
rs10156309	8q12.3	C	0.96	0.99	0.99
rs10124907	9p21.2	A	0.54	0.46	0.43
rs1332366	9q21.13	A	0.14	0.08	0.21
rs2472448	9q31.1	C	0.88	0.89	0.90
rs17133587	10p15.1	A	0.21	0.26	0.06
rs11027293	11p14.3	G	0.02	0.04	0.01
rs1893361	11q13.4	A	0.18	0.17	0.22
rs2884670	12p13.32	A	0.32	0.37	0.59
rs1352323	15q26.1	A	0.24	0.40	0.34
rs12597084	16p13.3	A	0.47	0.39	0.10
rs7187423	16q12.2	A	0.05	0.04	0.21
rs2061	17p12	A	0.07	0.10	0.21
rs7237244	18q11.2	C	0.98	0.89	0.99
rs12973620	19q13.43	C	0.18	0.25	0.12
rs7287939	22q13.2	C	0.07	0.06	0.02

*****Reference allele assigned as the allele coded 0 in the HapMap release 21 phased consensus haplotypes during genotype imputation in MACH
