# Supplementary figures and images for: Integrated genome-wide association, coexpression network, and expression single nucleotide polymorphism analysis identifies novel pathway in allergic rhinitis
Source: BMC Med Genomics. 2014 Aug 2;7:48. doi: 10.1186/1755-8794-7-48 (PMC4127082; doi:10.1186/1755-8794-7-48)

**Figure S2:** QQ plot for the GWAS meta-analysis of allergic rhinitis across ethnic groups

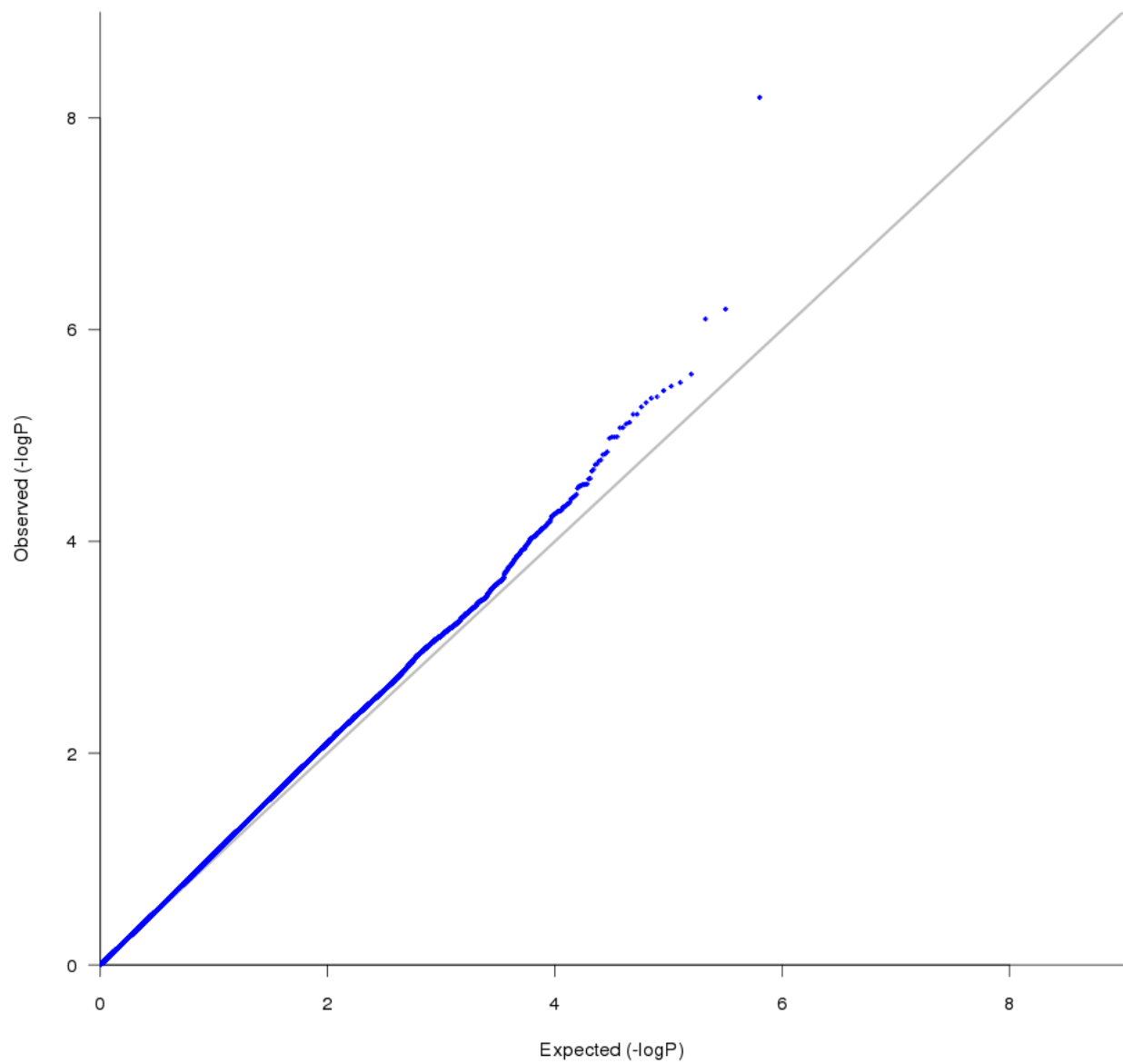

Supplement: Additional file 3: Figure S2 — QQ plot for the GWAS meta-analysis of allergic rhinitis across ethnic groups. [file 1755-8794-7-48-S3.pdf]
