# Supplementary material for: Integrated genome-wide association, coexpression network, and expression single nucleotide polymorphism analysis identifies novel pathway in allergic rhinitis
Source: BMC Med Genomics. 2014 Aug 2;7:48. doi: 10.1186/1755-8794-7-48 (PMC4127082; doi:10.1186/1755-8794-7-48)
Supplement: Additional file 4: Figure S3 — Regional associations for genome-wide significant loci (P value ≤ 5 × 10^−8^) in the GWAS of allergic rhinitis among Latinos. [file 1755-8794-7-48-S4.pdf]

Figure S3: Regional associations for genome-wide significant loci (P value ≤ 5 X 10^{-8}) in the GWAS of allergic rhinitis among Latinos

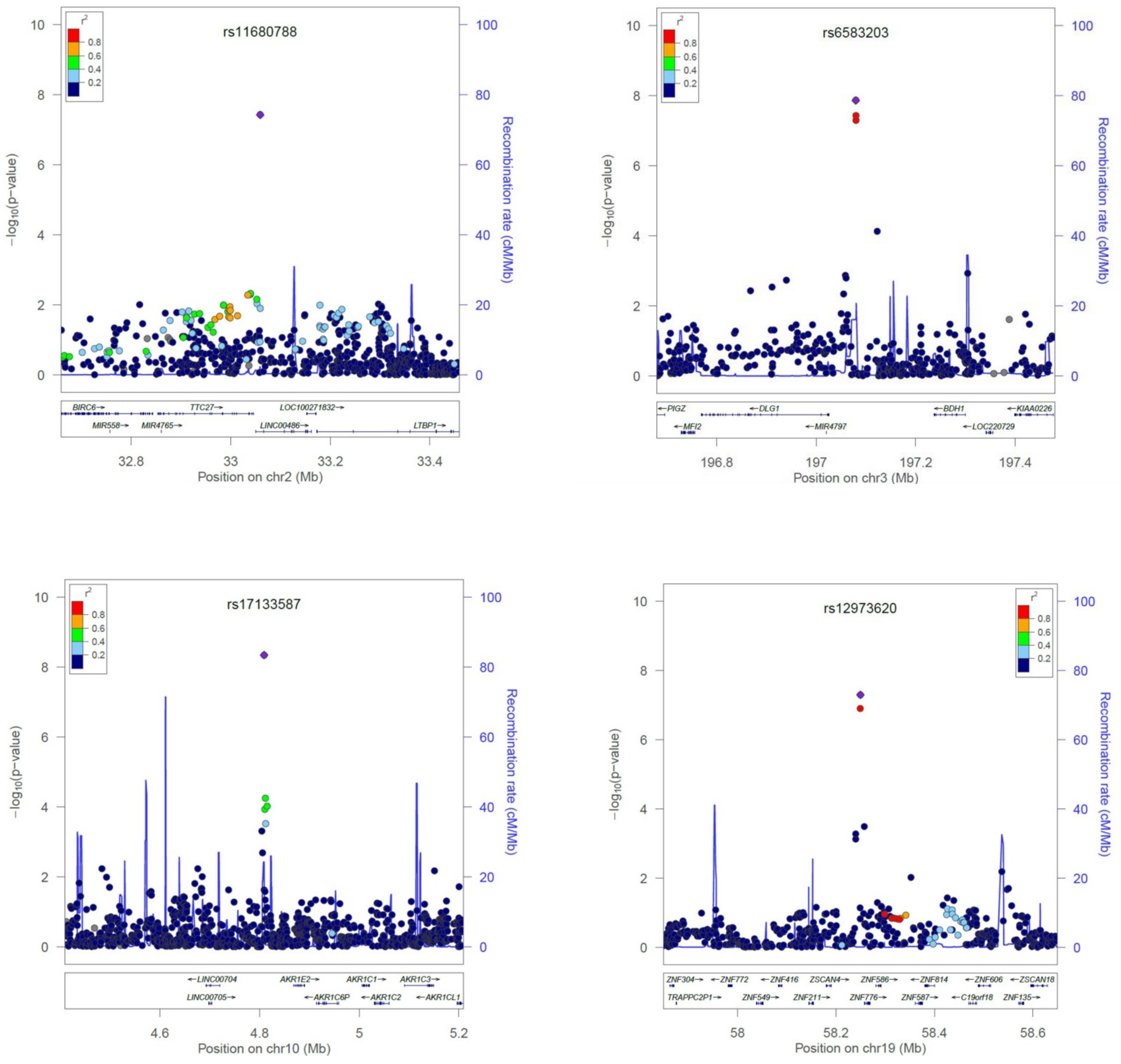

Reference genome for linkage disequilibrium calculations: hg19/1000 Genomes Mar 2012 AMR
