# Supplementary material for: Integrated genome-wide association, coexpression network, and expression single nucleotide polymorphism analysis identifies novel pathway in allergic rhinitis
Source: BMC Med Genomics. 2014 Aug 2;7:48. doi: 10.1186/1755-8794-7-48 (PMC4127082; doi:10.1186/1755-8794-7-48)
Supplement: Additional file 5: Table S2 — Mean Rsq values for genome-wide significant loci in the GWAS of allergic rhinitis among Latinos. [file 1755-8794-7-48-S5.pdf]

Table S2: Mean Rsq values for genome-wide significant loci in the GWAS of allergic rhinitis among Latinos

SNP	Rsq*
rs12973620	0.906
rs6583203	0.722
rs17133587	0.598
rs11680788	0.624

*estimates the squared correlation between imputed and true genotypes. Typically, a cut-off of 0.30 will flag most of the poorly imputed SNPs, but only a small number (<1%) of well imputed SNPs [59].
