# Supplementary material for: Integrated genome-wide association, coexpression network, and expression single nucleotide polymorphism analysis identifies novel pathway in allergic rhinitis
Source: BMC Med Genomics. 2014 Aug 2;7:48. doi: 10.1186/1755-8794-7-48 (PMC4127082; doi:10.1186/1755-8794-7-48)

Figure S4: Regional LD plots for genome-wide significant loci (P value ≤ 5 X 10-8) in the GWAS of allergic rhinitis among Latinos

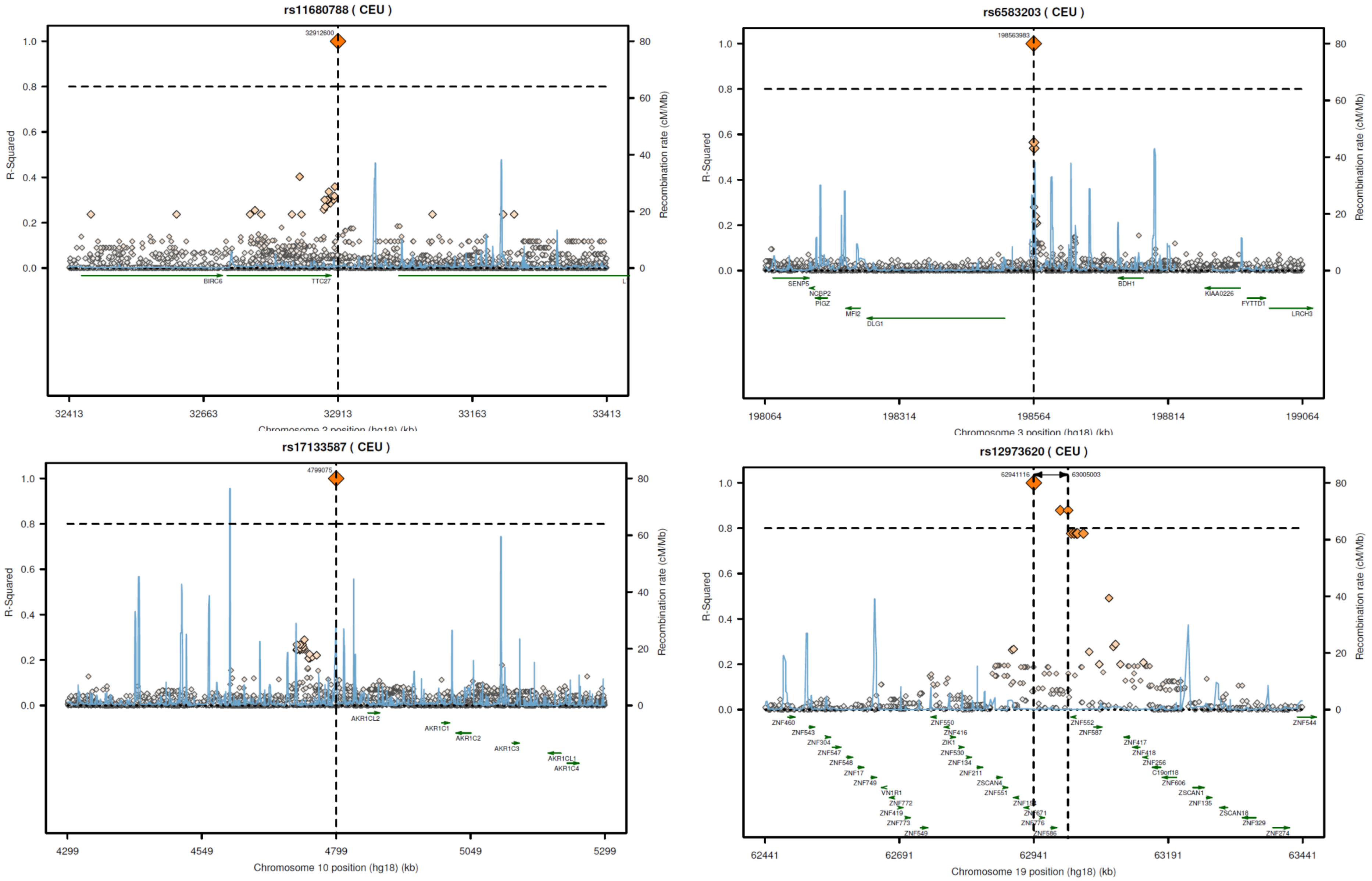

Supplement: Additional file 6: Figure S4 — Regional LD plots for genome-wide significant loci (P value ≤ 5 × 10^–8^) in the GWAS of allergic rhinitis among Latinos. [file 1755-8794-7-48-S6.pdf]
