# Supplementary material for: Integrated genome-wide association, coexpression network, and expression single nucleotide polymorphism analysis identifies novel pathway in allergic rhinitis
Source: BMC Med Genomics. 2014 Aug 2;7:48. doi: 10.1186/1755-8794-7-48 (PMC4127082; doi:10.1186/1755-8794-7-48)
Supplement: Additional file 7: Figure S5 — Regional associations for the genome-wide significant locus (P value 1.0 × 10^−8^) in the GWAS meta-analysis across ethnic groups. [file 1755-8794-7-48-S7.pdf]

Figure S5: Regional associations for the genome-wide significant locus (P value 1.0 X 10^{-8}) in the GWAS meta-analysis across ethnic groups

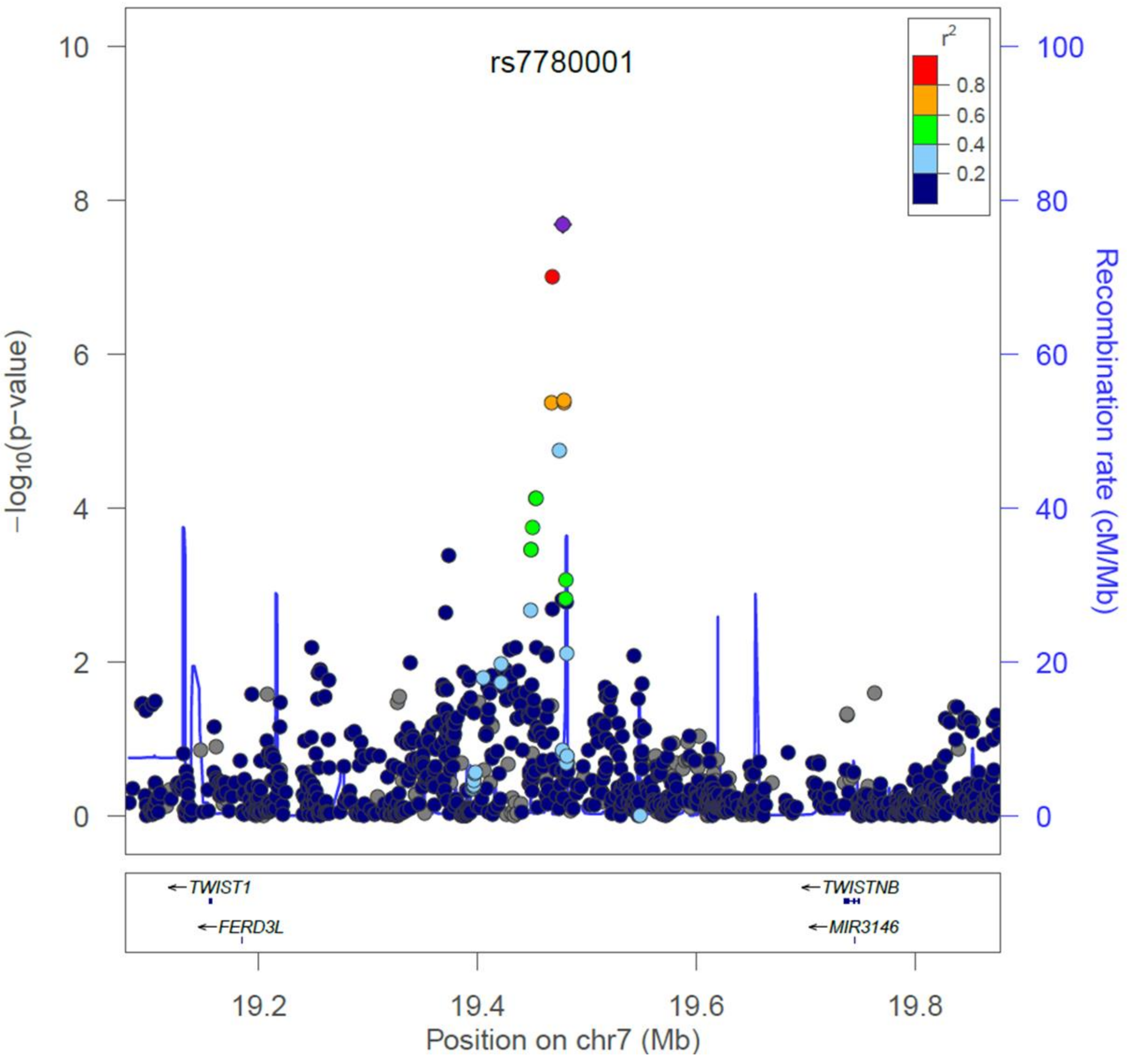

Reference genome for linkage disequilibrium calculations: hg19/1000 Genomes Mar 2012 EUR
