# Supplementary material for: Integrated genome-wide association, coexpression network, and expression single nucleotide polymorphism analysis identifies novel pathway in allergic rhinitis
Source: BMC Med Genomics. 2014 Aug 2;7:48. doi: 10.1186/1755-8794-7-48 (PMC4127082; doi:10.1186/1755-8794-7-48)
Supplement: Additional file 8: Table S3 — P values for association with allergic rhinitis for 17 loci identified in previous GWAS of allergic rhinitis. [file 1755-8794-7-48-S8.pdf]

Table S3: P values for association with allergic rhinitis for 17 loci identified in previous GWAS of allergic rhinitis

SNP	Location	Association reported by	Original study	Bunyavanich et al.	
				European American	Meta-Analysis
			P value	P value	P value
rs2155219	11q13.5	Ramasamy et al.[4]	3.8 x 10-8	0.22	0.06
rs2101521	4p14	Hinds et al.[9]	5.3 × 10−21	0.52	0.79
rs1438673	5q22.1	Hinds et al.[9]	2.3 × 10−20	0.31	0.51
rs2155219	11q13.5	Hinds et al.[9]	1.6 × 10−19	0.22	0.06
rs10189629	2q12.1	Hinds et al.[9]	1.8 × 10−16	0.48	**0.023**
rs6906021	6p21.32	Hinds et al.[9]	7.1 × 10−15	0.99	0.17
rs9266772	6p21.33	Hinds et al.[9]	3.2 × 10−12	0.25	0.11
rs7720838	5p13.1	Hinds et al.[9]	8.2 × 10−11	0.67	0.67
rs10497813	2q33.1	Hinds et al.[9]	6.1 × 10−10	**0.012**	**0.00099**
rs9860547	3q28	Hinds et al.[9]	1.2 × 10−9	0.83	0.94
rs7032572	9p24.1	Hinds et al.[9]	1.7 × 10−9	0.13	0.24
rs6021270	20q13.2	Hinds et al.[9]	6.9 × 10−9	0.87	0.43
rs9303280	17q12	Hinds et al.[9]	8.9 × 10−9	0.12	**0.0020**
rs17228058	15q22.33	Hinds et al.[9]	1.2 × 10−8	0.07	0.25
rs962993	10p14	Hinds et al.[9]	1.5 × 10−8	0.44	0.07
rs17388568	4q27	Hinds et al.[9]	3.9 × 10−8	**0.0030**	**0.0031**
rs1998359	14q21.1	Hinds et al.[9]	4.8 × 10−8	0.75	0.99

*P values with nominal significance (P value ≤ 0.05) in Bunyavanich et al. are bolded
