# Supplementary material for: Integrated genome-wide association, coexpression network, and expression single nucleotide polymorphism analysis identifies novel pathway in allergic rhinitis
Source: BMC Med Genomics. 2014 Aug 2;7:48. doi: 10.1186/1755-8794-7-48 (PMC4127082; doi:10.1186/1755-8794-7-48)
Supplement: Additional file 9: Supplementary Results 1 — Results for the GWAS of allergic rhinitis stratified by asthma status. [file 1755-8794-7-48-S9.pdf]

SUPPLEMENTARY RESULTS 1

GWAS of Allergic Rhinitis Stratified by Asthma Status

The GWAS of allergic rhinitis stratified by asthma status also showed ethnicity specific findings. Although there were no loci that met strict definitions of genome-wide significance (P value $\leq 5 \times 10^{-8}$), there were several loci with suggestive associations.

Among those without asthma (n= 2387; 661 with allergic rhinitis, 1726 without allergic rhinitis), variants on chromosome 5q23.2 and 21q21.1 had suggestive associations in European Americans, and a SNP on chromosome 21q22.12 had a suggestive association among African American/African Caribbeans (**Table S4**). rs7780001, which was associated with allergic rhinitis with genome-wide significance in the meta-analysis among all subjects, also had a suggestive association in the meta-analysis among those without asthma.

Among those with asthma (n= 3246; 2051 with allergic rhinitis, 1195 without allergic rhinitis), a locus near FGF20 on chromosome 8p22 had 14 SNPs (in high LD with one another) with suggestive associations with allergic rhinitis (P values 3.0 x 10-7 to 3.5 x 10-7) among African Americans/African Caribbeans only (**Figure S6**) .
