# Supplementary material for: Integrated genome-wide association, coexpression network, and expression single nucleotide polymorphism analysis identifies novel pathway in allergic rhinitis
Source: BMC Med Genomics. 2014 Aug 2;7:48. doi: 10.1186/1755-8794-7-48 (PMC4127082; doi:10.1186/1755-8794-7-48)
Supplement: Additional file 10: Table S4 — Results of the genome-wide association studies of allergic rhinitis among subjects without asthma. [file 1755-8794-7-48-S10.pdf]

Table S4: Results of the GWAS and meta-analysis for allergic rhinitis among subjects without asthma

SNP	Location	Allele	P value				OR (95% CI)				Allele Frequency		
			European American	Latino	African American	Meta - Analysis	European American	Latino	African American	Meta - Analysis	European American	Latino	African American
rs17152484	5q23.2	C	4.08E-07	0.56	0.32	1.17E-05	0.46 (0.15-0.76)	0.90 (0.53-1.22)	0.69 (0.00-1.43)	0.61 (0.38-0.83)	0.84	0.84	0.93
rs12520745	5q23.2	T	6.20E-07	0.63	0.94	1.26E-04	0.47 (0.18-0.77)	0.92 (0.55-1.27)	0.98 (0.47-1.49)	0.66 (0.54-0.82)	0.83	0.83	0.86
rs2606618	6q22.31	T	0.0011	0.0038	0.0080	3.87E-07	1.60 (1.32-1.88)	1.53 (1.24-2.58)	1.77 (1.25-2.20)	1.60 (1.33-1.92)	0.17	0.40	0.21
rs7780001	7p21.1	A	0.023	1.71E-04	0.0048	3.75E-07	0.70 (0.39-1.01)	0.55 (0.24-0.63)	0.55 (0.14-0.97)	0.61 (0.41-0.80)	0.87	0.80	0.76
rs2823048	21q21.1	A	8.19E-07	0.78	0.71	2.16E-04	0.52 (0.26-0.78)	1.06 (0.67-1.51)	0.92 (0.48-1.36)	0.69 (0.50-0.89)	0.81	0.88	0.81
rs2823053	21q21.1	A	8.39E-07	0.99	0.66	3.00E-04	0.51 (0.23-0.78)	1.00 (0.62-1.41)	0.93 (0.58-1.27)	0.71 (0.52-0.90)	0.82	0.84	0.55
rs2834629	21q22.12	T	0.93	0.92	5.24E-07	0.0057	1.03 (0.47-1.59)	0.96 (0.17-0.73)	0.17 (0.00-0.86)	0.58 (0.40-0.86)	0.91	0.92	0.88
