# Supplementary material for: Integrated genome-wide association, coexpression network, and expression single nucleotide polymorphism analysis identifies novel pathway in allergic rhinitis
Source: BMC Med Genomics. 2014 Aug 2;7:48. doi: 10.1186/1755-8794-7-48 (PMC4127082; doi:10.1186/1755-8794-7-48)
Supplement: Additional file 11: Figure S6 — Regional associations for the locus with suggestive associations in the GWAS of allergic rhinitis among African Americans/African Caribbeans with asthma. [file 1755-8794-7-48-S11.pdf]

Figure S6: Regional associations for the locus with suggestive associations in the GWAS of allergic rhinitis among African Americans/African Caribbeans with asthma

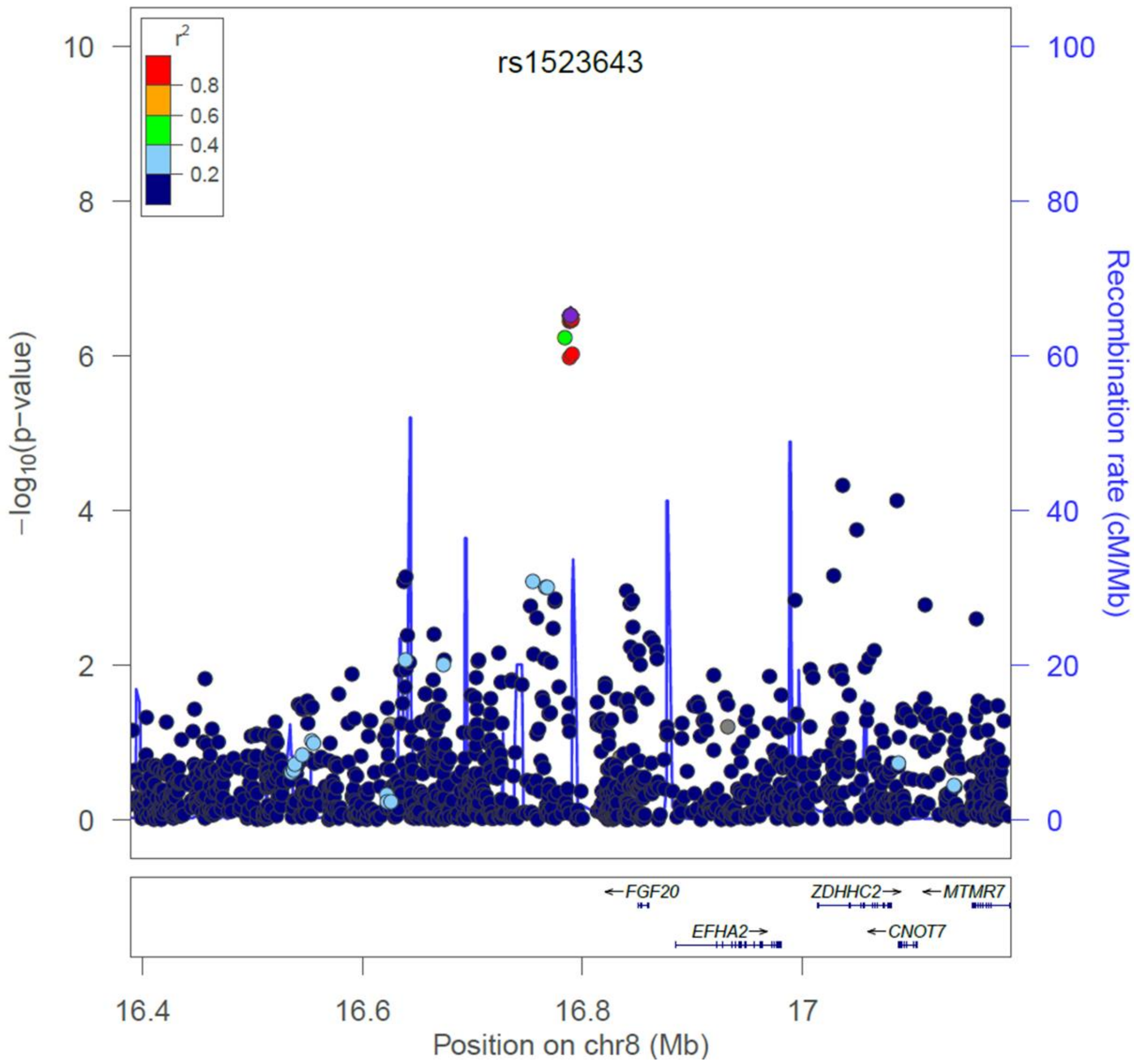

Reference genome for linkage disequilibrium calculations: hg19/1000 Genomes Mar 2012 AFR
