# Supplementary material for: Integrated genome-wide association, coexpression network, and expression single nucleotide polymorphism analysis identifies novel pathway in allergic rhinitis
Source: BMC Med Genomics. 2014 Aug 2;7:48. doi: 10.1186/1755-8794-7-48 (PMC4127082; doi:10.1186/1755-8794-7-48)
Supplement: Additional file 12: Table S5 — Sample composition of the stratified analysis according to asthma status. [file 1755-8794-7-48-S12.pdf]

Table S5: Sample composition of the stratified analysis according to asthma status

	Asthma		No Asthma	
	No Allergic Rhinitis	Allergic Rhinitis	No Allergic Rhinitis	Allergic Rhinitis
European American	488	623	674	249
Latino	545	1029	588	164
African American/African Caribbean	162	399	464	248
Total	1195	2051	1726	661
