# Supplementary material for: Integrated genome-wide association, coexpression network, and expression single nucleotide polymorphism analysis identifies novel pathway in allergic rhinitis
Source: BMC Med Genomics. 2014 Aug 2;7:48. doi: 10.1186/1755-8794-7-48 (PMC4127082; doi:10.1186/1755-8794-7-48)
Supplement: Additional file 13: Supplementary Results 2 — Random coexpression networks. [file 1755-8794-7-48-S13.pdf]

SUPPLEMENTARY RESULTS 2

Randomized coexpression networks

We generated multiple random coexpression networks where gene assignments were randomized (Random Networks 1-3), as well as random networks where the gene expression levels were randomized (Random Networks 4-6). The randomization of gene names preserved the correlation structure, resulting in the same network topology as we observed with the original, non-randomized data (**Figure S7**). However, enrichment of these modules for eSNPs followed a random distribution, in contrast to the strong eSNP enrichment patterns seen with the modules built with the non-randomized data (**Table 3**). In Random Networks 4-6, we randomized the entire matrix, thus destroying the correlation structure. The network topology was therefore changed (**Figure S7**), and in fact was no longer able to well fit a power law distribution. Distance structures became empty and no modules could be detected in any of these networks. This is consistent with previous reports that meaningful scale-free networks cannot be obtained when correlation structures are destroyed [24].
