# Supplementary material for: Integrated genome-wide association, coexpression network, and expression single nucleotide polymorphism analysis identifies novel pathway in allergic rhinitis
Source: BMC Med Genomics. 2014 Aug 2;7:48. doi: 10.1186/1755-8794-7-48 (PMC4127082; doi:10.1186/1755-8794-7-48)

Figure S7: Plots of scale free topology for coexpression networks constructed from randomized CD4+ gene expression data. Gene names were randomized in Random Networks 1-3. Gene expression values were randomized in Random Networks 4-6.

Random Network 1

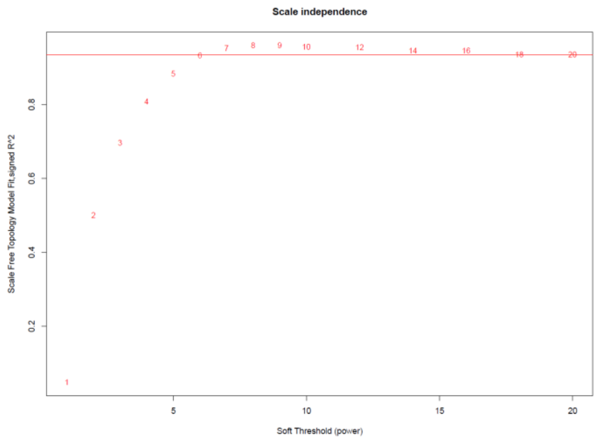

Random Network 2

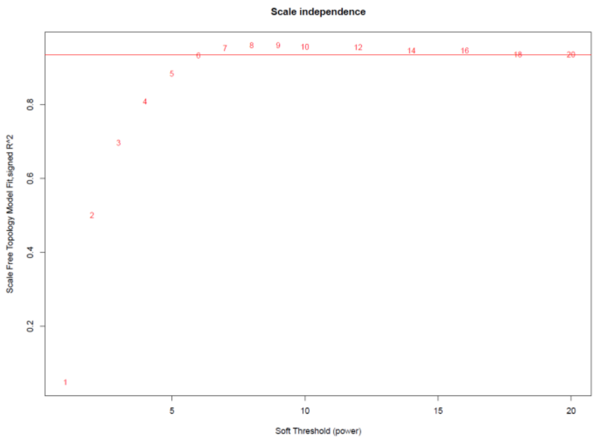

Random Network 3

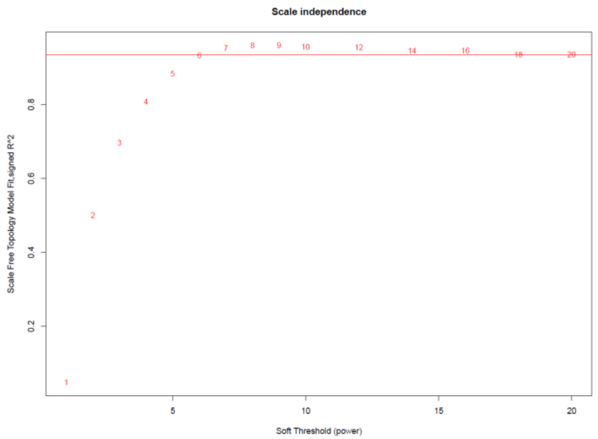

Random Network 4

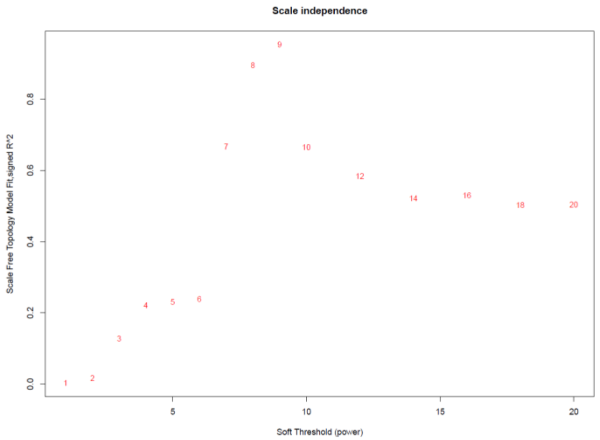

Random Network 5

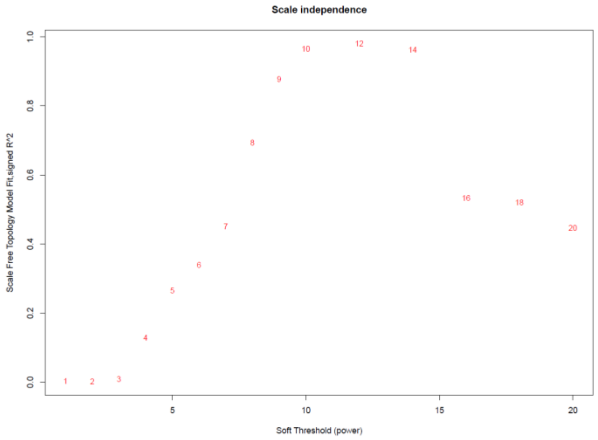

Random Network 6

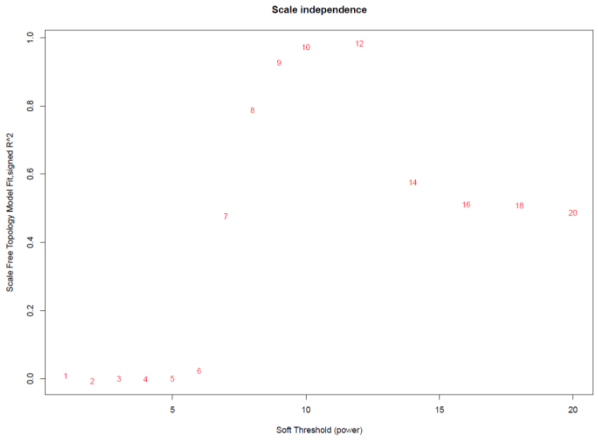

Supplement: Additional file 14: Figure S7 — Plots of scale free topology for coexpression networks constructed from randomized CD4+ gene expression data. Gene names were randomized in Random Networks 1–3. Gene expression values were randomized in Random Networks 4–6. [file 1755-8794-7-48-S14.pdf]
